# The added value of a micro-level ecological approach when mapping self-regulatory control processes and externalizing symptoms during adolescence: a systematic review

**DOI:** 10.1007/s00787-022-01972-1

**Published:** 2022-03-16

**Authors:** Sébastien Urben, Lauriane Constanty, Caroline Lepage, Joëlle Rosselet Amoussou, Julie Durussel, Fiorella Turri, Emilie Wouters, Ines Mürner-Lavanchy, Kerstin Jessica Plessen

**Affiliations:** 1 Division of Child and Adolescent Psychiatry, Department of Psychiatry, Lausanne University Hospital (CHUV), University of Lausanne, Lausanne, Switzerland; 2 Psychiatry Library, Education and Research Department, Lausanne University Hospital, University of Lausanne, Lausanne, Switzerland; 3 Unit of Child and Adolescent Forensic Psychiatry, Department of Psychiatry, Lausanne University Hospital (CHUV), Lausanne, Switzerland; 4 University Hospital of Child and Adolescent Psychiatry and Psychotherapy, University of Bern, Bern, Switzerland

**Keywords:** Self-regulation, Externalizing symptoms, Irritability, Adolescents, Emotion regulation, Heart rate variability, Respiratory sinus arrhythmia, Experience sampling methods, Ecological assessment

## Abstract

Deficits in self-regulatory control (SRC) represent a core characteristic of externalizing (EXT) symptoms (e.g., rule-breaking behavior or aggressive behaviors) in adolescents. This review aims to specify the added value of ecologically valid assessments at a micro-level when examining the associations between SRC and EXT symptoms in adolescents. This systematic review was reported according to the Preferred Reporting Items for Systematic Review and Meta-Analysis (PRISMA) 2020. The search strategy addressed the added value of (1) naturalistic assessment for the understanding of the relationship between (2) SRC and (3) EXT symptoms in (4) adolescents. We conducted comprehensive searches in bibliographic databases. An additional search was conducted in Google Scholar and supplementary studies were identified through backward and forward citation tracking. Twenty-four studies (*n* = 4071 adolescents) met the inclusion criteria. The methods used to assess naturalistic aspects included the experience sampling method (ecological momentary- or ambulatory assessment) and the time-course approach (i.e., real-time assessment of SRC processes referring to situations approximating real-life experience where SRC are to be engaged such as in frustrating situations). Micro-level ecological assessments, when mapping the intra-individual relationships between SRC processes and EXT symptoms over time in adolescents within their natural context (i.e., real world) of expression in real time, added a finer-grained observation alongside with a higher ecological validity. Micro-level approaches may enhance the understanding of the complex interplay between SRC and EXT symptoms in adolescence, especially in interventional studies, allowing for the acquisition of endpoints with a higher relevance for everyday functioning.

**Supplementary Information:**

The online version contains supplementary material available at 10.1007/s00787-022-01972-1.

## Background

### Self-regulatory processes and externalizing symptoms

Self-regulatory control (SRC) encompasses any intrinsic psychophysiological process allowing an individual to adapt their emotions, their thoughts and their behaviors to the ever-changing environment to achieve long-term goals [1]. So far, no consensus in the literature has been achieved regarding the definition of SRC. Thus, many terms have been used interchangeably (e.g., executive functions, effortful control, emotional regulation or self-control). Therefore, the umbrella term called SRC comprises heterogeneous processes. We adopted a broad and integrative perspective of SRC as we included different kinds of processes in the definition of SRC. In particular, we included cognitive (e.g., executive functions, effortful cognitive processes), emotional (e.g., emotion regulation, mood variability), physiological (e.g., heart rate variability—HRV or respiratory sinus arrhythmia—RSA) and social (e.g., parenting behaviors) processes (for a review of the definition of the processes and theoretical perspectives on SRC see [1]).

The failure of SRC and the emergence of antisocial or offending behaviors, also known as externalizing (EXT) symptoms (e.g., irritability, aggressive behaviors or conduct problems) are frequently related to one another [2–4] and may lead to severe and persistent delinquent or violent behaviors [5–9]. The breakdown of SRC processes may result in response disinhibition, impulsivity or risk taking [1], anger dysregulation [8] or physiological dysregulation [10].

EXT symptoms are more prevalent during adolescence [11, 12]. Indeed, adolescence refers to a critical period of heightened sensation seeking and risk taking [13] leading eventually to substance use, risky sexual behaviors and other problematic and/or challenging behaviors, such as EXT symptoms [14]. This pattern of behaviors may relate to the asynchrony of brain maturation within the subcortical brain structures, allowing emotional reactivity being fully developed before the prefrontal cortex reaches full maturity [15].

The evidence for associations between SRC processes and EXT symptoms in adolescents was mainly established in studies using group-level design and experimental procedures or longitudinal methodology indicating merely correlational relationships [2–4]. This prevents a fine-grained assessment of the dynamics in place between these processes. Moreover, the intra-individual variability can not be taken into account appropriately with such designs. More recent methodologies, however, grant access to the intra-individual level within the natural context (enhancing ecological validity), allowing a more in-depth observation and assessment of the specific processes linking SRC processes and EXT symptoms. Adopting a micro-time scale (e.g., on a day-to-day basis) within the naturalistic environment allows a precise understanding of the relationships between SRC and EXT symptoms at short-term (and contrary to the long-term trend observable with longitudinal studies) which might help to tailor intervention alleviating sufferance in daily-life. Moreover, it allows examining the patterns and relationships between specific events/stimuli and the subsequent behavior.

### Naturalistic assessment

The use of analyses on a micro-level may enhance our understanding of the nature and the variability of symptoms over time, both at a group and individual level compared to the standard or classical (or static) approach [16, 17]. Indeed, an ecological perspective on a micro-level allows examining the fluctuation and changes in affect, cognition, and behavior over time, known as dynamic processes at an intra-individual level [18] within the natural context (i.e., real world) of occurrence in “real time”. In addition, naturalistic methods do not suffer from recall bias due to retrospective assessment [19] and allow to assess of the temporal sequences among theoretically-linked constructs [20, 21]. Such contextual and dynamic assessments contribute to understand the complex interactions between SRC processes and EXT symptoms during adolescence within their natural context of expression. Such knowledge is of crucial importance when assessing outcomes of interventional studies or to develop ecological momentary interventions in a preventive approach [22].

At a micro-level (i.e., intra-individual assessment in real time), two complementary methods have been adopted in previous studies to examine the dynamics of the relationships between SRC processes and EXT symptoms: the experience sampling method (ESM; also called ecological momentary assessment or ambulatory assessment) and the time-course approach. In particular, ESM allows to capture intra-individual variability through repeated assessments within a natural (or ecological) environment [23]. The time-course approach (i.e., “in situation” or real-time assessment), on the other hand, allows to capture subtle changes in SRC in real time within situations where SRC processes must be engaged.

Consequently, we aimed to determine the relevant literature to estimate the added value of micro-level ecological approaches (e.g., ESM, time-course approach/real-time assessment) for the understanding of associations between the failure of SRC processes (i.e., emotional, cognitive, social or physiological) and EXT symptoms in adolescents.

## Methods

### Procedure

The reporting of this systematic review was guided by the standards of the Preferred Reporting Items for Systematic Review and Meta-Analysis (PRISMA) 2020 [24, 25]. The review protocol is available on PROSPERO (#CRD42020192629). The inclusion criteria are studies (1) conducted with adolescent samples (mean age between 12 and 18), (2) assessing SRC processes (i.e., cognitive, emotional, social or physiological) through a naturalistic or ecological methodology such as ESM (i.e., multiple assessments per day to apprehend intra-individual variability and avoid recall bias or time-course), real-time assessment or “in situation” (i.e., placing the individual in situation approximating real-life experience where they have to self-regulate [e.g., stressing or frustrating situation] and observe the dynamic of changes in SRC processes); (3) measuring EXT symptoms (e.g., aggressive behaviors, conduct problems, irritability/anger dysregulation) or the categorical disorders encompassing EXT symptoms (e.g., conduct disorders, intermittent explosive disorder, oppositional defiant disorder); (4) manuscript published in English or French (this criterion has been applied when screening the studies). As the aim of the review was to focus on EXT symptoms, we adopted a transdiagnostic approach within externalizing disorders. However, studies focusing mainly on attention-deficit/hyperactivity disorder (ADHD), substance use, sexual offending, autism spectrum disorder or intellectual disabilities were not included as are specific areas of research leading to abundant literature, which are beyond the scope of this review. The reason being that EXT symptoms in these psychopathological difficulties are more related to other components (e.g., neurodevelopmental difficulties). Two independent reviewers (SU and JD) conducted all steps of the process (e.g., study selection, data extraction). We discussed choices that differed between reviewers to achieve a consensus. We lead a network analysis with the Igraph package [26] running on R v.3.6.0 [27] to illustrate the relationships between the SRC and psychopathological symptoms. We refrained from performing a meta-analysis, due to the study designs, the measures and the specific assessments being too heterogeneous to conduct a reliable pooled analysis.

### Search strategy

**Fig. 1:**
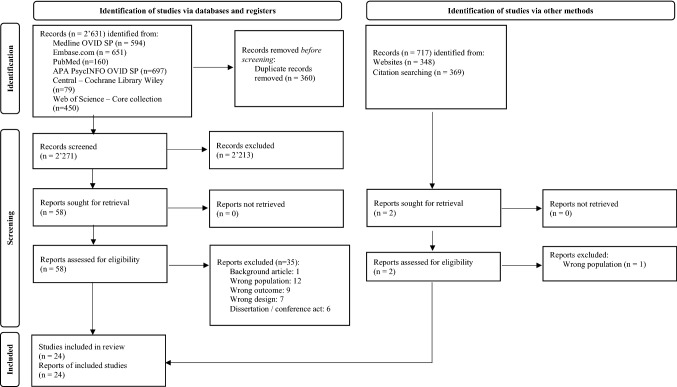
Flow diagram

A medical librarian conducted the search (JRA) using the fours search concepts (i.e., adolescent, SRC, EXT, naturalistic assessment), by consulting the following bibliographic databases on March 4, 2021: Embase.com, Medline Ovid, PubMed, APA PsycINFO Ovid, Web of Science Core Collection, Cochrane Library Wiley, Open Grey, ProQuest Dissertations & Theses A&I, Dart Europe, LISSA, and SantéPsy (Ascodocpsy). Clinical trials were searched in CENTRAL Cochrane Library Wiley, ClinicalTrials.gov and the World health organization international clinical trials registry platform. An additional search was conducted in Google Scholar. All searches were done without language or date restrictions. Additional records were identified through citation tracking, using backward and forward citation strategies. Forward citation chaining was performed in the Web of Science Core collection and Google Scholar. Figure 1 displayed the flow diagram following the latest PRISMA guidelines [24]. The supplementary file 1 describes the full search strategies.

We screened the title and abstract of identified studies for possible inclusion (*k* = 2271), which led to *k* = 60 studies selected for full-text screening of which 24 studies met inclusion criteria defined in the protocol for the study (see Table 1).

**Table 1:** Summary of the included studies

Authors	Origin	*N*	Male	Female	Age	Sample	Ecological aspects
Beauchaine, Katkin [31]	USA	59	59	0	13.4	EXT disorders	Time-course
Byrd, Vine [51]	USA	162	86	76	12.0	EXT disorders	Mix
Cui, Zhang [29]	USA	57	31	26	13.2	Light behavioral and emotional difficulties	Time-course
Cui, Morris [44]	USA	206	91	115	13.4	Light behavioral and emotional difficulties	Time-course
de Ridder, Pihet [42]	Switzerland	55	45	10	14.8	EXT disorders	ESM
Diamond, Fagundes [52]	USA	110	56	54	14.0	Healthy adolescents	Mix
Gonzalez-Gadea, Herrera [49]	Colombia	46	46	0	16.4	Offenders	Time-course
Gunlicks-Stoessel and Powers [30]	USA	80	31	49	16.8	Healthy adolescents	Time-course
Klahr, Rueter [47]	USA	1199	540	659	14.0	Healthy adolescents	Time-course
Kuhn, Ahles [45]	USA	134	64	70	12.6	Healthy adolescents	Time-course
Maciejewski, Keijsers [36]	Netherland	482	275	207	13.0	Light behavioral and emotional difficulties	ESM
Moore, Hubbard [37]	USA	144	64	80	13.6	Healthy adolescents	ESM
Odgers and Russell [32]	USA	151	79	72	13.0	Light behavioral and emotional difficulties	ESM
Pihet, De Ridder [43]	Switzerland	103	80	23	14.8	EXT disorders	ESM
Rende, Slomkowski [33]	USA	120	53	67	16.5	Healthy adolescents	ESM
Rothenberg, Di Giunta [41]	Italy	103	55	48	16.8	Healthy adolescents	ESM
Santamaria-Garcia, Ibanez [50]	Colombia	65	65	0	17.1	Offenders	Time-course
Schneiders, Nicolson [38]	Netherland	131	72	59	12.9	Healthy adolescents	ESM
Silk, Steinberg [39]	USA	152	73	79	13.9	Healthy adolescents	ESM
Thomas, Jain [48]	USA	40	18	22	15.0	Healthy adolescents	Time-course
Uink, Modecki [40]	Australia	206	80	126	14.6	Light behavioral and emotional difficulties	ESM
Uink, Modecki [34]	Australia	108	34	74	14.7	Light behavioral and emotional difficulties	ESM
Ungvary, McDonald [46]	USA	58	36	22	14.1	Healthy adolescents	Time-course
Vannucci, Ohannessian [35]	USA	100	60	40	15.1	Healthy adolescents	ESM

*EXT* externalizing; *ESM* experience sampling method

### Critical appraisal

To assess the bias (e.g., selection of the participants, sample size justification, drop-out analyses, statistical method, ethical consideration, the role of funding sources) and, thus, the quality of the retrieved studies, we used the AXIS instrument [28]. This instrument listed 20 items to assess critically the quality of an observational study ranging from study aims, methodology, results but also funding sources and ethical aspects (for details see Table S1 and Figure S1).

### Study categorization

We extracted the relevant information from each study. Firstly, we characterized the samples as following: (a) Healthy adolescents (i.e., healthy individuals), (b) Subtle behavioral and emotional difficulties (i.e., individuals at risk of embarking on a criminal career—e.g., adolescents with emotional or behavioral difficulties, student considered disruptive by the teacher or with academic difficulties); (c) Offenders (i.e., youths incarcerated in a juvenile facility or under probationary period following the commission of offences), (d) EXT disorders (i.e., adolescents with severe externalizing disorders). Secondly, we described the specific process of SRC (i.e., cognition, emotion, social, physiology) and the psychopathological symptoms (i.e., aggressive behaviors, irritability/anger dysregulation, conduct problems, offending, impulsivity, callous-unemotional traits, ADHD symptoms, substance use, and as comorbid symptoms also depressive symptoms or anxiety) examined in the respective study (see Table 2).

**Table 2:** Descriptive

		Total (*n* = 24)
Origin	USA	15
	Australia	2
	Switzerland	2
	The Netherland	2
	Italy	1
	Colombia	2
Sample	Healthy adolescents	12
	Subtle behavioral and emotional difficulties	6
	Offenders	2
	EXT disorders	4
Study design	Correlates	13
	Time course	4
	Case–control studies	4
	Cohort study	3
SR processes*	Cognition	5
	Emotion	19
	Social	11
	Physiology	8
Symptoms*	Aggressive behaviors	20
	Conduct problems	13
	Irritability/anger	9
	Offending	9
	Impulsivity	3
	Callous-unemotional traits	2
	ADHD symptoms	2
	Substance use	5
	Depressive symptoms	12
	Anxiety	9

*SR* self-regulation; *EXT* externalizing; *ADHD* attention-deficit/hyperactivity disorder

^*^Studies may include more than one process/symptom

## Results

### Descriptive

Twelve studies (50%) used ESM methodology whereas 10 studies (42%) adopted a time-course approach (or “in situation” assessment of SRC processes). Finally, two studies (8%) used a mixed methodology combining ESM and time-course approaches. All studies were published during the last 20 years and the majority, *k* = 17 (71%) since 2016 (see Table 2).

From the 24 studies considered in this review, we observed that 15 are reported to have a high degree of ethnic diversity including Caucasians, Asians, Latinos, African Americans, Europeans, Hispanics, East Indians, Hawaiians, Pacific Islanders, Aborigines, and Maoris samples*.* Whereas, the total number of participants in the 24 publications included in the review were *n* = 4071 with an equivalent proportion of male and female participants (see Table 1). The mean age was 14.0 years (ranging from 10 to 20 years). Fifty percent of the studies included healthy participants, 25% participants with light behavioral and emotional difficulties, 17% participants with EXT disorders and 8% were qualified as offenders. The studies were conducted in North America (*k* = 15, 68% of the participants), Europe (*k* = 5, 21% of the participants), Colombia (*k* = 2, 3% of the participants) and Australia (*k* = 2, 8% of the participants).

### Critical appraisal

The critical appraisal of the studies, using the AXIS instrument, showed that most of the studies displayed appropriate quality with no major flaws regarding the aim, the method used, the funding sources or the ethical aspects (see supplementary Table S1 and Figure S1). Nevertheless, only three studies provided a justification of the sample size by a power analysis or at least some arguments [29–31]. Moreover, many studies lacked a description of non-responders (and dropouts), and an assessment of the selection bias for the results. Therefore, the selection bias and therewith non-responders in the study represent important limitations of previous studies.

### Network analysis: SRC × EXT symptoms

**Fig. 2:**
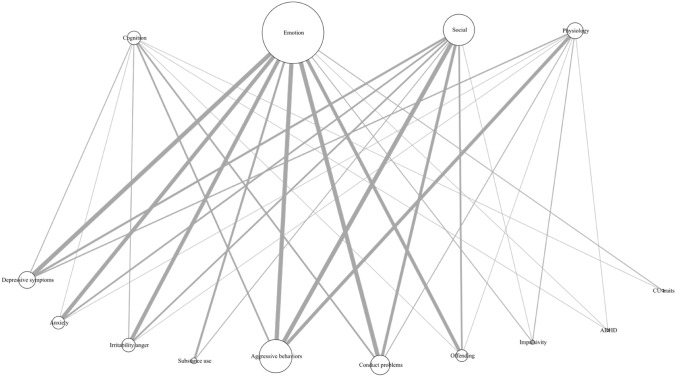
Network analysis examining the relationships between self-regulatory processes and symptoms. Upper part: Self-regulatory control processes and lower part: Psychopathological symptoms. The size of the vertices are relative to the number of studies. The edge widths are relative to the number of studies (up to 10, for illustration purpose). *ADHD* attention deficit hyperactivity disorder symptoms; *CU* callous-unemotional

We performed a network analysis examining the associations between specific SRC processes (i.e., cognitive, emotion, social and physiology) and psychopathological symptoms (see Fig. 2).

The majority of studies assessed the association between regulation of emotion and aggressive behaviors (*k* = 17), followed by the association of emotion and conduct problems (*k* = 10) and by the association of emotion and irritability/anger dysregulation (*k* = 9). Moreover, 10 studies examined the relationship between social SRC (e.g., parenting behaviors) processes and aggressive behaviors. Fewer studies focused on cognitive (*k* = 5) and physiological (*k* = 8) SRC processes alongside with EXT symptoms such as callous-unemotional traits (*k* = 2), and impulsivity (*k* = 3).

### Results summary

All studies examined EXT symptoms or behaviors as an outcome and studied the predictive value of SRC processes (i.e., specified in the function of the target of regulation, namely cognition, emotion, social or physiology). Notice that the majority of the study examined emotion-related processes (e.g., mood variability) which are components of the umbrella term of SRC, including both emotional reactivity and regulation, which are balanced under the supervision of SRC. Internalizing symptoms were studied as covariates or controlled variables in 12 studies.

#### ESM studies

Within ESM studies, we observed that four studies [32–35] assessed the importance of the surroundings/environment for negative emotionality and psychopathology, while six studies [36–41] focused on mood variability. Finally, two studies [42, 43] examined adolescents with severe EXT behaviors. All studies fixed triggers to respond either at a priori planned moment during the day or randomly, ranging from 1 to 9 assessments per day during 5–30 consecutive days.

##### Surrounding/environment impact

A study observed (ESM protocol: 3x/day for 30 consecutive days) that witnessing violence (i.e., social component of SRC) in young adolescents was associated with psychopathological symptoms on the same day, but also with depressive symptoms, irritability (i.e., emotional component of SRC) as well as conduct problems on the next day [32]. In another study (ESM protocol: prompted 2x/hours excluding school and sleep hours for 6 consecutive days) antisocial behavior (i.e., rule-breaking), which was related to negative emotionality, was more likely to occur when siblings were at a friend’s house [33]. Along the same lines, a study (ESM protocol: 1x/day at evening for 14 consecutive days) reported a bidirectional association between conflict with friends and emotionality (e.g., anger, sadness, anxiety) at a daily level [35]. Moreover, a study (ESM protocol: 5x/day for 7 days) observed an impact of gender and the social context (i.e., peer relationships) on emotional reactivity to daily stressors. Indeed, spending time with peers in the hours after a negative event blunted the emotional reaction (e.g., sadness, worry or jealousy), especially in girls [34].

##### Daily variability of negative emotion

A longitudinal study (from 13 to 20 years) reported, that the vast majority of youths (88%) showed a decrease in mood variability during adolescence (ESM protocol: 1x/day for three weeks), which was related to a decline in depressive and EXT symptoms suggesting that the decrease of mood variability throughout adolescence is a protective factor from psychopathologies [36]. Likewise, a study (ESM protocol: 3x/day for 15 consecutive days) revealed that sadness dysregulation is related to the expression of depressive symptoms, whereas, anger dysregulation is associated with aggressive behaviors on a particular day [41]. Similarly, mood reactivity towards everyday life events (ESM protocol: 9x/day for 5 consecutive days) influenced the relationships between daily stress and mental health in adolescents [38]. In particular, daily negative events were related to lower positive mood and higher negative emotions (i.e., depressed mood, anxiety, and irritation). Notice that the social context had an important impact. Indeed, events within the familial environment triggered more likely sadness whereas negative events within the school environment are more likely to increase anxiety. Moreover, a study (ESM protocol: 6–7x/day for 7 consecutive days) highlighted that non-adaptive emotion regulation strategies (i.e., denial or rumination) leading to experiencing negative emotions in daily-life were related to higher degree of depressive symptoms and problematic behavior [39]. Another study (ESM protocol: 12 consecutive evenings) reported that reactive aggression was related to emotional over-reactivity to negative events whereas proactive aggression was related to blunted affectivity [37]. In particular, reactive aggression was related to higher daily anger, more variability of anger during the day and heightened anger to negative events as well as to lower level of happiness but higher reactivity of happiness to positive events. In contrast, proactive aggression was not related to daily emotions except that it was related to less variability in happiness during the day. Finally, adolescents with high EXT symptoms reported (ESM protocol: 5x/days for 7 days) higher emotional reactivity (greater increase in sadness, anger, jealousy and loneliness) relative to a stressing event, although these adolescents did not report being confronted with more stressful events [40].

##### ESM with adolescents with severe EXT disorders

In a study, ESM (4x/day for 8 days) was successfully applied to adolescents with severe EXT disorders revealing its feasibility and satisfactory compliance of the participants as well as reliability to capture intra-individual variability of impulsivity, negative affectivity and antisocial behavior [43]. Likewise, adolescents with conduct disorders as well as high callous-unemotional traits performed as accurately as adolescents with low callous-unemotional traits in recognizing emotions (e.g., anger, distress) expressed by staff members in their institution (ESM protocol: 4x/day for 8 days). However, adolescents overestimated the intensity of the emotion [42].

#### Time-course approach / real-time assessment

Five studies [29, 31, 44–46] focused on physiological (i.e., RSA: influence of respiration on heart rate) or emotional SRC measures during stressful tasks and three other studies [30, 47, 48] used parent-adolescent discussion tasks eliciting conflicts and their associations with EXT symptoms. Finally, two other studies featured on contextual information in emotion processing in offenders [49, 50].

##### Physiological markers in stressful tasks

Studies using the time-course approach examined the reactivity of physiological SRC markers (i.e., RSA) during stressful tasks such as viewing a film portraying a case of bullying [29], a film showing an escalation in the conflict between peers [31] or the angry event discussion task [44]. Moreover, changes in physiological SRC (i.e., RSA suppression) measured during an emotion-eliciting task, buffered the associations between neighborhood violence and aggressive behaviors [29]. Likewise, stronger physiological SRC markers buffered the relationships between trait impulsivity and EXT symptoms measured prospectively, at a 6-month follow-up study [45]. Finally, baseline low level of RSA was related to victimization by peers and to reactive aggression in adolescents [46].

##### Parent-adolescents conflict

A mild increase in the level of negative emotions (and not the mean level during a conflict negotiation task between adolescents and their mother), in typically developing adolescents, across the negotiation was predictive of adolescents’ adjustments (i.e., internalizing and EXT symptoms) [30]. Moreover, low physiological SRC markers mediated the relationships between parent–adolescent conflict and adolescent propensity to take the risk [48]. Parent–adolescent conflict (assessed through both perspectives) were associated with antisocial behaviors in adopted and non-adopted adolescents [47].

##### Contextual information

A study on empathy within real-life scenarios in adolescent offenders compared with typically developing adolescents, indicated impairment in social contextual processing in adolescent offenders [49]. Likewise, adolescent offenders were observed to overestimate information from emotional body language and contextual surrounding in emotion perception [50]. This association was related to differences in gray matter volumes of brain regions involved in body perception (fusiform gyrus), emotional information (cingulate cortex, superior temporal gyrus), contextual integration (precuneus, superior temporal gyrus), and motor resonance (cerebellum, supplementary motor area) [50].

#### Methodologies combing the two approaches

Two studies [51, 52] used a time-course approach coupled with an ESM protocol revealing three main findings. First, individual differences in physiological SRC markers (measured during a stress-inducing task) moderated the sensitivity to family environmental characteristics (i.e., mother’s internalizing symptoms as well as family structure) measured through an ESM protocol [52]. Second, the interaction between low baseline RSA and higher RSA reactivity to parent–adolescents conflict was related to complex emotion dysregulation (i.e., shame, guilt, loneliness, emptiness). Third, higher baseline RSA was associated with behavioral dysregulation, whereas higher RSA withdrawal to parent–adolescent conflict was linked to higher simple emotion dysregulation (i.e., sadness, anger, nervousness, stress) [51].

## Discussion

The studies identified by the present systematic review confirm the previous knowledge on the importance of psychophysiological markers to understand SRC processes [53], as well as the role of SRC played in the emergence and maintenance of EXT symptoms [2–4]. Subsequently, they add value to the characterization of lived experience (i.e., ESM, real-time assessment/time-course approach). Moreover, they articulate the within-person variability alongside the inter-person differences, examine the temporal dynamic, reduce recall bias and allow to understand the role of the context (i.e., real world). More specifically, the reviewed studies stress the contribution of micro-level approaches (i.e., ambulatory assessment, ESM, real-time assessment or time-course approach) for determining the importance of the context, the impact of real-life experiences (e.g., role of conflicts), the role of mood variability as well as the dynamic of SRC changes during stressful situations.

### How were SRC processes studied within naturalistic context?

ESM protocols [2, 36, 37] allow coupling the complex interactions of emotional SRC processes, in particular negative affectivity or mood variability, with the surrounding or environment (i.e., social aspects of SRC) on EXT. Furthermore, the underlying physiological dynamics of emotional SRC processes were also successfully studied within a time-course approach [30, 49]. By contrast, physiological SRC markers were only studied through time-course approach and revealed the pattern of RSA changes (i.e., suppression and recovery) and its usefulness to predict future behaviors but also to buffer adversity (e.g., neighborhood violence) [29, 44–46]. The cognitive components of SR processes were scarcely examined through subjective assessment in ESM protocols [39, 42], but more often using the time-course approach, which allowed to understand the variability in cognitive performance related to emotional reactivity [31]. This may be due to the difficulty of performing a reliable assessment of cognitive performance outside the lab, as well as the technical difficulties of assuring multiple cognitive assessments in the natural environment.

### Added values of the micro-level naturalistic approaches

One of the advantages of using an ESM approach, is to categorize lived experiences (i.e., real time) within the natural environment (i.e., real world), and as such it enhances ecological validity (compared to laboratory or static assessment) [54, 55]. This approach provides insights into the intra-individual level of emotionality and psychopathology and into the nature and temporal sequence of SRC processes in real time within a natural context. Thus, it enhances the understanding of SRC functioning and its links with EXT symptoms. In particular, the ESM approach consists of frequent, repeated assessment of thoughts, cognition, experiences and behaviors in the naturalistic environment (i.e., real world). The time-course approach puts the individual in a laboratory setting generating anger/frustration, for instance, which enables the observation of changes in the relationships between psychophysiological SRC processes and EXT symptoms in a direct observational setting (i.e., in real time) and, thus, is ideally suited to measure treatment effects in a non-biased way, independent of the informant. The main important difference from the traditional approach is that SRC processes can be observed in live or “in situation” instead of assessing the representation of the functioning of SRC by the individual. Despite being conducted in a laboratory environment, this approach avoids social desirability bias as well as artificial assessment. Moreover, this approach was observed to yield information more relevant to predict future adjustment problems than the conventional (i.e., static assessment) one [44].

Moreover, the daily life approach (or ESM) stressed the role of the natural environment and experiences such as conflicts [32–35] as well as the daily mood variability [36–40] in the expression of EXT symptoms. In particular, ESM studies allow to capture the dynamic and changing nature of the relationships between emotions experienced [41] or regulated [39] in everyday life and EXT symptoms in adolescents. Moreover, they allow a better understanding of these relationships by specifying the emotions experienced related to negative daily events, which provide important clues to understand the emergence of psychopathological symptoms [38]. In addition, ESM studies allow understanding the risk [32] or protective [34] role of the environment in the expression of EXT symptoms. Finally, two studies [51, 52] coupling these approaches (i.e., ESM and in the situation) revealed the importance and usefulness of combining these approaches to have an in-depth understanding of the role of SRC processes in EXT symptoms.

In addition, the ESM approach reduced recall bias leading to more accurate assessments of the processes at play [54, 56]. Moreover, the micro-level approach allowed articulating intra-individual or within-person variability with inter-individual differences [57] in the relationships between SRC (e.g., mood variability) and EXT symptoms. Indeed, through advanced statistical approaches, the micro-level approach improved the possibility to provide causal inferences [56, 58] between the SRC dysfunctions and the expression of EXT symptoms permitting more specific theorization of this interplay. Therefore, we can study the precursor and outcomes through such an approach, which enables us to adopt a short-term longitudinal design, and in turn provides the possibility to assess how dysfunction of SRC processes may cause EXT symptoms.

### Clinical relevance and implications

From a clinical point of view, as emotion (and concomitant physiological) reactivity and regulation depend on situational demands (e.g., conflicts, stressful events) and context [59], micro-level approach is particularly relevant in this domain. Indeed, such an approach permits to control for contextual demands and, thus, provides a fine-grained observation of emotional or physiological SRC expression in real time and in real life situation (ESM) or in approximating real-world experiences (time-course approach). This allows us to enhance our understanding of the at-risk situations and may help us to tailor the therapeutical interventions [56]. Likewise, these approaches might allow developing more specific targets for prevention and intervention and measurement of their effectiveness as well as help to acquire endpoints with a higher relevance for everyday functioning in interventional studies.

### Future perspectives

Study protocols including other objective measures will permit a deeper understanding of the role played by the SRC processes in EXT symptoms. For instance, functional Magnetic Resonance Imaging (fMRI), functional near-infrared spectroscopy (fNIRS) or electroencephalography (EEG) measures may contribute to the understanding of the neural underpinnings of SRC time-courses and their specific relationships with the expression of EXT symptoms. The inclusion of other mobile and wearable measures, such as actigraphy is of high importance as they allow observing interrelationships between sleep, physical activity, subjective level of energy and emotions in real time [60]. Likewise, a recent scoping review stressed the importance of mobile and wearable measures in children and adolescents with depressive disorders [61]. Thus, wearable measures may as well be applied to contribute to the understanding of EXT symptoms in future research. Moreover, cognitive aspects of SRC, such as inhibitory control might be also useful aspects that should be studied in future studies within ESM protocols [62]. Finally, to apprehend the social SR processes within the time-course approach, one can manipulate the presence of peers while performing the task, such as in the procedure adopted by Gardner and Steinberg [63] which revealed the influence of peers on risk taking during adolescence. In that line, mobile technologies offer new opportunities and novel insights in the understanding of developmental psychopathologies [64]. Future studies should also consider event-based sampling methods or, in other terms, assessment triggered by a specific event such as the expression of symptoms like aggression or antisocial behaviors. This was never done in previous studies and might help to deepen the understanding of the relationships between SRC and EXT symptoms.

### Limitations

We limited this systematic review to adolescents; thus further reviews should focus on children or adults. Moreover, the systematic review was limited to literature published in English and French. We may therefore have missed some information published in other languages, however, this is unlikely to taint the global observed picture, as we did not limit the search in the first place. In a transdiagnostic approach, we excluded studies focusing specifically on ADHD, substance use, sexual offending behaviors, autism spectrum disorder or intellectual disabilities and results thus pertain to persons suffering from external problems in a dimensional definition. Regarding the heterogeneity in the study design and operationalization of the relationships between SR and EXT symptoms, we did not conduct a quantitative meta-analysis, which will be helpful in the future when the methodology will be more harmonious and thus comparable. The categorization of the symptoms is challenging regarding the conceptual overlap between aggressive behaviors, impulsivity, anger dysregulation and conduct problems (see for instance [65]). However, we based our assessment on the instrument used in each study to represent, at best, the concepts that were measured.

### Conclusions

This systematic review describes studies using micro-level naturalistic approaches to assess the relationships of SRC processes and EXT symptoms in adolescents. Such approaches yield insights beyond the static and group-level knowledge and, thus, allow to apprehend intra-individual variability (i.e., ESM), but also to assess SRC processes in real time during their use (i.e., time-course) within their context of expression (i.e., real world). Therefore, these approaches contribute to developing more in-depth knowledge of the link between SRC processes and EXT symptoms, which will allow developing more specific targets for prevention and intervention and measurement of their effectiveness. In addition, the micro time scale allows us to observe the time window when SRC are in play and to comprehend how dysfunction of these abilities is related to the expression of EXT symptoms. Moreover, micro-level naturalistic approaches may help to acquire endpoints with a higher relevance for everyday functioning in interventional studies. However, regarding the heterogeneity of the methodology and study designs used in previous studies, further studies should help to standardize this valuable approach and to develop a gold standard to enhance the generalization and comparability of the results.

### Supplementary Information

Below is the link to the electronic supplementary material.

Supplementary file1 (DOCX 26 KB)

Supplementary file2 (DOCX 72 KB)
