# Supplementary material for: The added value of a micro-level ecological approach when mapping self-regulatory control processes and externalizing symptoms during adolescence: a systematic review
Source: Eur Child Adolesc Psychiatry. 2022 Mar 16;32(12):2387–97. doi: 10.1007/s00787-022-01972-1 (PMC10682160; doi:10.1007/s00787-022-01972-1)
Supplement: Supplementary file 1 — Supplementary file1 (DOCX 26 KB) [file 787_2022_1972_MOESM1_ESM.docx]

Supplementary file 1: The added-value of a micro-level ecological approach when mapping self-regulatory control processes and externalizing symptoms during adolescence: A systematic review / Sébastien Urben, Lauriane Constanty, Caroline Lepage, Joëlle Rosselet Amoussou, Julie Durussel, Emilie Wouters, Ines Mürner-Lavanchy & Kerstin Jessica Plessen

Search strategy

Note:

- The strategy consists of two search equations combined with the Boolean operator OR. The first equation looks for the concept of "ecological momentary assessment" with externalizing disorders in adolescence. The second equation combines ecological approaches with externalized disorders in adolescence, and self-regulatory processes.
- The research strategies were peer reviewed by another information specialist prior to execution.

Bibliographic database search strategies

All searches were conducted without language or date restrictions.

**Embase.com**

Last Content Update, 4 Mar 2021 01:36:56 GMT

623 references found, March 4, 2021

(('ecological momentary assessment'/de OR ("ecologic* momentary assess*" OR EMA):ab,ti,kw) AND ('externalizing disorder'/exp OR 'oppositional defiant disorder'/de OR 'intermittent explosive disorder'/de OR 'conduct disorder'/de OR 'antisocial personality disorder'/de OR 'behavior disorder'/de OR 'impulse control disorder'/exp OR 'disruptive behavior'/exp OR 'impulsiveness'/de OR 'inhibition (psychology)'/exp OR 'sensation seeking'/de OR 'high risk behavior'/de OR 'irritability'/de OR 'aggression'/exp OR 'theft'/de OR 'antisocial behavior'/exp OR 'offender'/de OR (externalizing OR externalising OR ODD OR ((oppositional OR defiant) NEXT/1 disorder*) OR "intermittent explosive disorder" OR "conduct disorder*" OR ((sociopath* OR psychopath* OR antisocial OR dyssocial) NEXT/1 (behav* OR personalit*)) OR sociopathy OR ((aberrant OR deviant OR disturb*) NEXT/1 behav*) OR (behav* NEXT/1 (aberration OR disorder* OR disturbance OR crisis)) OR runaway OR elopement OR "impulse control disorder*" OR "conduct disorder*" OR ((disruptive OR problem* OR dysfunctional) NEAR/3 behav*) OR problembehav* OR impulsiv* OR impulsigenic OR disinhibition OR inhibition OR "sensation seek*" OR risk-taking OR "risk behav*" OR "risky behav*" OR irritability OR "irritable mood*" OR ((defiant OR hostile) NEXT/1 behav*) OR aggress* OR counteraggress* OR anger OR "angry outbursts" OR provocation* OR deceitful* OR theft* OR stealing* OR ((antisocial OR anti-social OR asocial* OR deviant) NEAR/3 (behav* OR conduct* OR personalit* OR reaction*)) OR "social behav* disorder*" OR misbehav* OR misconduct* OR delinquency OR delinquent* OR offender* OR callous-unemotional OR "callous and unemotional"):ab,ti,kw) **AND** ('juvenile'/de OR 'adolescent'/exp OR 'young adult'/de OR 'adolescence'/exp OR 'child psychiatry'/de OR 'juvenile delinquency'/de OR (adolescent* OR adolescence OR preadolescen* OR pre-adolescen* OR teen* OR juvenile OR youth* OR "young adult*"):ab,ti,kw) NOT ([animals]/lim NOT [humans]/lim)) **OR** (('phenomenology'/de OR ("experience sampling*" OR phenomenolog*):ab,ti,kw OR (("real time" OR time-course OR ecological OR in-situ OR naturalistic OR real-world OR "daily life" OR "everyday life"):ab,ti,kw AND ('questionnaire'/exp OR 'interview'/exp OR 'self report'/de OR 'self evaluation'/de OR (experience* OR experiment* OR measur* OR questionnaire* OR interview* OR scale* OR self-report* OR "self evaluation*" OR "self appraisal*" OR diary OR diaries OR assess* OR environment* OR approach*):ab,ti,kw)) OR ((daily OR everyday) NEAR/3 (experience* OR experiment* OR measur* OR questionnaire* OR interview* OR scale* OR evaluation* OR appraisal* OR report* OR assess* OR diary OR diaries)):ab,ti,kw) AND ('externalizing disorder'/exp OR 'oppositional defiant disorder'/de OR 'intermittent explosive disorder'/de OR 'conduct disorder'/de OR 'antisocial personality disorder'/de OR 'behavior disorder'/de OR 'impulse control disorder'/exp OR 'disruptive behavior'/exp OR 'impulsiveness'/de OR 'inhibition (psychology)'/exp OR 'sensation seeking'/de OR 'high risk behavior'/de OR 'irritability'/de OR 'aggression'/exp OR 'theft'/de OR 'antisocial behavior'/exp OR 'offender'/de OR (externalizing OR externalising OR ODD OR ((oppositional OR defiant) NEXT/1 disorder*) OR "intermittent explosive disorder" OR "conduct disorder*" OR ((sociopath* OR psychopath* OR antisocial OR dyssocial) NEXT/1 (behav* OR personalit*)) OR sociopathy OR ((aberrant OR deviant OR disturb*) NEXT/1 behav*) OR (behav* NEXT/1 (aberration OR disorder* OR disturbance OR crisis)) OR runaway OR elopement OR "impulse control disorder*" OR "conduct disorder*" OR ((disruptive OR problem* OR dysfunctional) NEAR/3 behav*) OR problembehav* OR impulsiv* OR impulsigenic OR disinhibition OR inhibition OR "sensation seek*" OR risk-taking OR "risk behav*" OR "risky behav*" OR irritability OR "irritable mood*" OR ((defiant OR hostile) NEXT/1 behav*) OR aggress* OR counteraggress* OR anger OR "angry outbursts" OR provocation* OR deceitful* OR theft* OR stealing* OR ((antisocial OR anti-social OR asocial* OR deviant) NEAR/3 (behav* OR conduct* OR personalit* OR reaction*)) OR "social behav* disorder*" OR misbehav* OR misconduct* OR delinquency OR delinquent* OR offender* OR callous-unemotional OR "callous and unemotional"):ab,ti,kw) AND ('self control'/de OR 'executive function'/de OR 'metacognition'/exp OR 'ego'/de OR 'autoregulation'/exp OR 'heart rate'/de OR 'heart rate variability'/de OR 'respiratory sinus arrhythmia'/de OR 'vagus tone'/de OR 'pressoreceptor reflex'/de OR 'autonomic nervous system'/de OR 'adrenergic system'/de OR (self-control OR "self manag*" OR "executive function*" OR "cognitive control" OR ((executive OR proactive OR reactive) NEXT/1 control) OR "effortful control" OR metacognition OR metacognitive OR meta-cognit* OR ((emotion* OR affect) NEXT/1 regulation) OR "emotional control" OR (control NEXT/3 emotion*) OR "ego control" OR "ego undercontrol" OR "ego resiliency" OR "ego depletion" OR autoregulation OR self-regulat* OR homeostasis OR ((cardiac OR heart) NEXT/1 (frequenc* OR rate*)) OR "respiratory sinus arrhythmia" OR "vagus tone" OR "vagal tone" OR vagotonus OR "vagus nerve tone" OR baroreflex* OR "pressoreceptor reflex" OR "baroceptor reflex" OR "baroreceptor reflex" OR "pressor reflex" OR "pressure reflex" OR "baroreceptor reflex" OR ((autonom* OR vegetative) NEXT/3 system) OR ((adrenergic OR sympath* OR orthosympath*) NEXT/3 (mechanism OR system))):ab,ti,kw) AND ('juvenile'/de OR 'adolescent'/exp OR 'young adult'/de OR 'adolescence'/exp OR 'child psychiatry'/de OR 'juvenile delinquency'/de OR (adolescent* OR adolescence OR preadolescen* OR pre-adolescen* OR teen* OR juvenile OR youth* OR "young adult*"):ab,ti,kw) NOT ([animals]/lim NOT [humans]/lim))

**Medline Ovid SP**

Ovid MEDLINE(R) and Epub Ahead of Print, In-Process, In-Data-Review & Other Non-Indexed Citations and Daily 1946 to February 25, 2021

547 references found, February 26, 2021

(("Ecological Momentary Assessment"/ OR ("ecologic* momentary assess*" OR EMA).ab,ti,kf.) AND ("Attention Deficit and Disruptive Behavior Disorders"/ OR "Conduct Disorder"/ OR "Disruptive, Impulse Control, and Conduct Disorders"/ OR "Antisocial Personality Disorder"/ OR "Problem Behavior"/OR "Impulsive Behavior"/ OR "Inhibition, Psychological"/ OR "Irritable Mood"/ OR exp "Aggression"/ OR "Theft"/ OR "Social Behavior Disorders"/ OR "Juvenile Delinquency"/ OR "Child Behavior Disorders"/ OR "Risk-Taking"/ OR (externalizing OR externalising OR ODD OR ((oppositional OR defiant) ADJ1 disorder*) OR "intermittent explosive disorder" OR "conduct disorder*" OR ((sociopath* OR psychopath* OR antisocial OR dyssocial) ADJ1 (behav* OR personalit*)) OR sociopathy OR ((aberrant OR deviant OR disturb*) ADJ1 behav*) OR (behav* ADJ1 (aberration OR disorder* OR disturbance OR crisis)) OR runaway OR elopement OR "impulse control disorder*" OR "conduct disorder*" OR ((disruptive OR problem* OR dysfunctional) ADJ3 behav*) OR problembehav* OR impulsiv* OR impulsigenic OR disinhibition OR inhibition OR "sensation seek*" OR risk-taking OR "risk behav*" OR "risky behav*" OR irritability OR "irritable mood*" OR ((defiant OR hostile) ADJ1 behav*) OR aggress* OR counteraggress* OR anger OR "angry outbursts" OR provocation* OR deceitful* OR theft* OR stealing* OR ((antisocial OR anti-social OR asocial* OR deviant) ADJ3 (behav* OR conduct* OR personalit* OR reaction*)) OR "social behav* disorder*" OR misbehav* OR misconduct* OR delinquency OR delinquent* OR offender* OR callous-unemotional OR "callous and unemotional").ab,ti,kf.) AND ("Adolescent"/ OR "Young Adult"/ OR "Adolescent Psychiatry"/ OR "Psychology, Adolescent"/ OR "Juvenile Delinquency"/ OR (adolescent* OR adolescence OR preadolescen* OR pre-adolescen* OR teen* OR juvenile OR youth* OR "young adult*").ab,ti,kf.) NOT (exp animals/ NOT humans.sh.)) OR ((("experience sampling*" OR phenomenolog*).ab,ti,kf. OR (("real time" OR time-course OR ecological OR in-situ OR naturalistic OR real-world OR "daily life" OR "everyday life").ab,ti,kf. AND (exp "Surveys and Questionnaires"/ OR "Interviews as Topic"/ OR "Diagnostic Self Evaluation"/ OR "Self-Assessment"/ OR (experience* OR experiment* OR measur* OR questionnaire* OR interview* OR scale* OR self-report* OR "self evaluation*" OR "self appraisal*" OR diary OR diaries OR assess* OR environment* OR approach*).ab,ti,kf.)) OR ((daily OR everyday) ADJ3 (experience* OR experiment* OR measur* OR questionnaire* OR interview* OR scale* OR evaluation* OR appraisal* OR report* OR assess* OR diary OR diaries)).ab,ti,kf.) AND ("Attention Deficit and Disruptive Behavior Disorders"/ OR "Conduct Disorder"/ OR "Disruptive, Impulse Control, and Conduct Disorders"/ OR "Antisocial Personality Disorder"/ OR "Problem Behavior"/ OR "Impulsive Behavior"/ OR "Inhibition, Psychological"/ OR "Irritable Mood"/ OR exp "Aggression"/ OR "Theft"/ OR "Social Behavior Disorders"/ OR "Juvenile Delinquency"/ OR "Child Behavior Disorders"/ OR "Risk-Taking"/ OR (externalizing OR externalising OR ODD OR ((oppositional OR defiant) ADJ1 disorder*) OR "intermittent explosive disorder" OR "conduct disorder*" OR ((sociopath* OR psychopath* OR antisocial OR dyssocial) ADJ1 (behav* OR personalit*)) OR sociopathy OR ((aberrant OR deviant OR disturb*) ADJ1 behav*) OR (behav* ADJ1 (aberration OR disorder* OR disturbance OR crisis)) OR runaway OR elopement OR "impulse control disorder*" OR "conduct disorder*" OR ((disruptive OR problem* OR dysfunctional) ADJ3 behav*) OR problembehav* OR impulsiv* OR impulsigenic OR disinhibition OR inhibition OR "sensation seek*" OR risk-taking OR "risk behav*" OR "risky behav*" OR irritability OR "irritable mood*" OR ((defiant OR hostile) ADJ1 behav*) OR aggress* OR counteraggress* OR anger OR "angry outbursts" OR provocation* OR deceitful* OR theft* OR stealing* OR ((antisocial OR anti-social OR asocial* OR deviant) ADJ3 (behav* OR conduct* OR personalit* OR reaction*)) OR "social behav* disorder*" OR misbehav* OR misconduct* OR delinquency OR delinquent* OR offender* OR callous-unemotional OR "callous and unemotional").ab,ti,kf.) AND ("Self-Control"/ OR "Executive Function"/ OR "Metacognition"/ OR exp "Ego"/ OR "Homeostasis"/ OR "Baroreflex"/ OR exp "Heart Rate"/ OR "Autonomic Nervous System"/ OR (self-control OR "self manag*" OR "executive function*" OR "cognitive control" OR ((executive OR proactive OR reactive) ADJ1 control) OR "effortful control" OR metacognition OR metacognitive OR meta-cognit* OR ((emotion* OR affect) ADJ1 regulation) OR "emotional control" OR (control ADJ3 emotion*) OR "ego control" OR "ego undercontrol" OR "ego resiliency" OR "ego depletion" OR autoregulation OR self-regulat* OR homeostasis OR ((cardiac OR heart) ADJ1 (frequenc* OR rate*)) OR "respiratory sinus arrhythmia" OR "vagus tone" OR "vagal tone" OR vagotonus OR "vagus nerve tone" OR baroreflex* OR "pressoreceptor reflex" OR "baroceptor reflex" OR "baroreceptor reflex" OR "pressor reflex" OR "pressure reflex" OR "baroreceptor reflex" OR ((autonom* OR vegetative) ADJ3 system) OR ((adrenergic OR sympath* OR orthosympath*) ADJ3 (mechanism OR system))).ab,ti,kf.) AND ("Adolescent"/ OR "Young Adult"/ OR "Adolescent Psychiatry"/ OR "Psychology, Adolescent"/ OR "Juvenile Delinquency"/ OR (adolescent* OR adolescence OR preadolescen* OR pre-adolescen* OR teen* OR juvenile OR youth* OR "young adult*").ab,ti,kf.) NOT (exp animals/ NOT humans.sh.))

**PubMed**

Search limited to non-MEDLINE articles (NOT medline[sb])

128 references found, February 26, 2021

(("ecologic momentary assess*"[tiab] OR "ecological momentary assess*"[tiab] OR EMA[tiab]) AND (externalizing[tiab] OR externalising[tiab] OR ODD[tiab] OR ((oppositional[tiab] OR defiant[tiab]) AND disorder*[tiab]) OR "intermittent explosive disorder"[tiab] OR conduct disorder*[tiab] OR ((sociopath*[tiab] OR psychopath*[tiab] OR antisocial[tiab] OR dyssocial[tiab]) AND (behav*[tiab] OR personalit*[tiab])) OR sociopathy[tiab] OR ((aberrant[tiab] OR deviant[tiab] OR disturb*[tiab]) AND behav*[tiab]) OR (behav*[tiab] AND (aberration[tiab] OR disorder*[tiab] OR disturbance[tiab] OR crisis[tiab])) OR runaway[tiab] OR elopement[tiab] OR impulse control disorder*[tiab] OR conduct disorder*[tiab] OR ((disruptive[tiab] OR problem*[tiab] OR dysfunctional[tiab]) AND behav*[tiab]) OR impulsiv*[tiab] OR impulsigenic[tiab] OR disinhibition[tiab] OR inhibition[tiab] OR sensation seek*[tiab] OR risk-taking[tiab] OR risk behav*[tiab] OR risky behav*[tiab] OR irritability[tiab] OR irritable mood*[tiab] OR ((defiant[tiab] OR hostile[tiab]) AND behav*[tiab]) OR aggress*[tiab] OR counteraggress*[tiab] OR anger[tiab] OR "angry outbursts"[tiab] OR provocation*[tiab] OR deceitful*[tiab] OR theft*[tiab] OR stealing*[tiab] OR ((antisocial[tiab] OR anti-social[tiab] OR asocial*[tiab] OR deviant[tiab]) AND (behav*[tiab] OR conduct*[tiab] OR personalit*[tiab] OR reaction*[tiab])) OR social behavior disorder*[tiab] OR social behaviour disorder*[tiab] OR social behavioral disorder*[tiab] OR social behavioural disorder*[tiab] OR misbehav*[tiab] OR misconduct*[tiab] OR delinquency[tiab] OR delinquent*[tiab] OR offender*[tiab] OR callous-unemotional[tiab] OR "callous and unemotional"[tiab]) AND (adolescent*[tiab] OR adolescence[tiab] OR preadolescen*[tiab] OR pre-adolescen*[tiab] OR teen*[tiab] OR juvenile[tiab] OR youth*[tiab] OR young adult*[tiab]) NOT medline[sb]) OR (("experience sampling*"[tiab] OR phenomenolog*[tiab] OR (("real time"[tiab] OR time-course[tiab] OR ecological[tiab] OR in-situ[tiab] OR naturalistic[tiab] OR real-world[tiab] OR "daily life"[tiab] OR "everyday life"[tiab]) AND (experience*[tiab] OR experiment*[tiab] OR measur*[tiab] OR questionnaire*[tiab] OR interview*[tiab] OR scale*[tiab] OR self-report*[tiab] OR "self evaluation*"[tiab] OR "self appraisal*"[tiab] OR diary[tiab] OR diaries[tiab] OR assess*[tiab] OR environment*[tiab] OR approach*[tiab])) OR ((daily[tiab] OR everyday[tiab]) AND (experience*[tiab] OR experiment*[tiab] OR measur*[tiab] OR questionnaire*[tiab] OR interview*[tiab] OR scale*[tiab] OR evaluation*[tiab] OR appraisal*[tiab] OR report*[tiab] OR assess*[tiab] OR diary[tiab] OR diaries[tiab]))) AND (externalizing[tiab] OR externalising[tiab] OR ODD[tiab] OR ((oppositional[tiab] OR defiant[tiab]) AND disorder*[tiab]) OR "intermittent explosive disorder"[tiab] OR conduct disorder*[tiab] OR ((sociopath*[tiab] OR psychopath*[tiab] OR antisocial[tiab] OR dyssocial[tiab]) AND (behav*[tiab] OR personalit*[tiab])) OR sociopathy[tiab] OR ((aberrant[tiab] OR deviant[tiab] OR disturb*[tiab]) AND behav*[tiab]) OR (behav*[tiab] AND (aberration[tiab] OR disorder*[tiab] OR disturbance[tiab] OR crisis[tiab])) OR runaway[tiab] OR elopement[tiab] OR impulse control disorder*[tiab] OR conduct disorder*[tiab] OR ((disruptive[tiab] OR problem*[tiab] OR dysfunctional[tiab]) AND behav*[tiab]) OR impulsiv*[tiab] OR impulsigenic[tiab] OR disinhibition[tiab] OR inhibition[tiab] OR sensation seek*[tiab] OR risk-taking[tiab] OR risk behav*[tiab] OR risky behav*[tiab] OR irritability[tiab] OR irritable mood*[tiab] OR ((defiant[tiab] OR hostile[tiab]) AND behav*[tiab]) OR aggress*[tiab] OR counteraggress*[tiab] OR anger[tiab] OR "angry outbursts"[tiab] OR provocation*[tiab] OR deceitful*[tiab] OR theft*[tiab] OR stealing*[tiab] OR ((antisocial[tiab] OR anti-social[tiab] OR asocial*[tiab] OR deviant[tiab]) AND (behav*[tiab] OR conduct*[tiab] OR personalit*[tiab] OR reaction*[tiab])) OR social behavior disorder*[tiab] OR social behaviour disorder*[tiab] OR social behavioral disorder*[tiab] OR social behavioural disorder*[tiab] OR misbehav*[tiab] OR misconduct*[tiab] OR delinquency[tiab] OR delinquent*[tiab] OR offender*[tiab] OR callous-unemotional[tiab] OR "callous and unemotional"[tiab]) AND (self-control[tiab] OR self manag*[tiab] OR executive function*[tiab] OR "cognitive control"[tiab] OR ((executive[tiab] OR proactive[tiab] OR reactive[tiab]) AND control[tiab]) OR "effortful control"[tiab] OR metacognition[tiab] OR metacognitive[tiab] OR meta-cognit*[tiab] OR ((emotion*[tiab] OR affect[tiab]) AND regulation[tiab]) OR "emotional control"[tiab] OR (control[tiab] AND emotion*[tiab]) OR "ego control"[tiab] OR "ego undercontrol"[tiab] OR "ego resiliency"[tiab] OR "ego depletion"[tiab] OR autoregulation[tiab] OR self-regulat*[tiab] OR homeostasis[tiab] OR ((cardiac[tiab] OR heart[tiab]) AND (frequenc*[tiab] OR rate*[tiab])) OR "respiratory sinus arrhythmia"[tiab] OR "vagus tone"[tiab] OR "vagal tone"[tiab] OR vagotonus[tiab] OR "vagus nerve tone"[tiab] OR baroreflex*[tiab] OR "pressoreceptor reflex"[tiab] OR "baroceptor reflex"[tiab] OR "baroreceptor reflex"[tiab] OR "pressor reflex"[tiab] OR "pressure reflex"[tiab] OR "baroreceptor reflex"[tiab] OR ((autonom*[tiab] OR vegetative[tiab]) AND system[tiab]) OR ((adrenergic[tiab] OR sympath*[tiab] OR orthosympath*[tiab]) AND (mechanism[tiab] OR system[tiab]))) AND (adolescent*[tiab] OR adolescence[tiab] OR preadolescen*[tiab] OR pre-adolescen*[tiab] OR teen*[tiab] OR juvenile[tiab] OR youth*[tiab] OR young adult*[tiab]) NOT medline[sb])

**APA PsycINFO Ovid SP**

APA PsycInfo 1806 to February Week 4 2021

635 references found, March 4, 2021

((ecological momentary assessment/ OR ("ecologic* momentary assess*" OR EMA).mp.) AND (externalizing symptoms/ OR oppositional defiant disorder/ OR exp impulse control disorders/ OR conduct disorder/ OR antisocial personality disorder/ OR explosive disorder/ OR exp behavior problems/ OR behavior disorders/ OR behavioral disinhibition/ OR "inhibition (personality)"/ OR juvenile delinquency/ OR impulsiveness/ OR irritability/ OR exp aggressive behavior/ OR aggressiveness/ OR theft/ OR antisocial behavior/ OR risk taking/ OR sensation seeking/ OR (externalizing OR externalising OR ODD OR ((oppositional OR defiant) ADJ1 disorder*) OR "intermittent explosive disorder" OR "conduct disorder*" OR ((sociopath* OR psychopath* OR antisocial OR dyssocial) ADJ1 (behav* OR personalit*)) OR sociopathy OR ((aberrant OR deviant OR disturb*) ADJ1 behav*) OR (behav* ADJ1 (aberration OR disorder* OR disturbance OR crisis)) OR runaway OR elopement OR "impulse control disorder*" OR "conduct disorder*" OR ((disruptive OR problem* OR dysfunctional) ADJ3 behav*) OR problembehav* OR impulsiv* OR impulsigenic OR disinhibition OR inhibition OR "sensation seek*" OR risk-taking OR "risk behav*" OR "risky behav*" OR irritability OR "irritable mood*" OR ((defiant OR hostile) ADJ1 behav*) OR aggress* OR counteraggress* OR anger OR "angry outbursts" OR provocation* OR deceitful* OR theft* OR stealing* OR ((antisocial OR anti-social OR asocial* OR deviant) ADJ3 (behav* OR conduct* OR personalit* OR reaction*)) OR "social behav* disorder*" OR misbehav* OR misconduct* OR delinquency OR delinquent* OR offender* OR callous-unemotional OR "callous and unemotional").mp.) AND (adolescent attitudes/ OR adolescent psychiatry/ OR adolescent psychology/ OR adolescent psychopathology/ OR adolescent psychotherapy/ OR exp juvenile delinquency/ OR (adolescent* OR adolescence OR preadolescen* OR pre-adolescen* OR teen* OR juvenile OR youth* OR "young adult*").mp.)) OR ((phenomenology/ OR interpretative phenomenological analysis/ OR ("experience sampling*" OR phenomenolog*).mp. OR (("real time" OR time-course OR ecological OR in-situ OR naturalistic OR real-world OR "daily life" OR "everyday life").mp. AND (exp questionnaires/ OR exp interviews/ OR exp psychophysiological measures/ OR attitude measures/ OR measurement/ OR self-report/ OR self-evaluation/ OR "experiences (events)"/ OR journal writing/ OR (experience* OR experiment* OR measur* OR questionnaire* OR interview* OR scale* OR self-report* OR "self evaluation*" OR "self appraisal*" OR diary OR diaries OR assess* OR environment* OR approach*).mp.)) OR ((daily OR everyday) ADJ3 (experience* OR experiment* OR measur* OR questionnaire* OR interview* OR scale* OR evaluation* OR appraisal* OR report* OR assess* OR diary OR diaries)).mp.) AND (externalizing symptoms/ OR oppositional defiant disorder/ OR exp impulse control disorders/ OR conduct disorder/ OR antisocial personality disorder/ OR explosive disorder/ OR exp behavior problems/ OR behavior disorders/ OR behavioral disinhibition/ OR "inhibition (personality)"/ OR exp juvenile delinquency/ OR impulsiveness/ OR irritability/ OR exp aggressive behavior/ OR aggressiveness/ OR theft/ OR antisocial behavior/ OR risk taking/ OR sensation seeking/ OR (externalizing OR externalising OR ODD OR ((oppositional OR defiant) ADJ1 disorder*) OR "intermittent explosive disorder" OR "conduct disorder*" OR ((sociopath* OR psychopath* OR antisocial OR dyssocial) ADJ1 (behav* OR personalit*)) OR sociopathy OR ((aberrant OR deviant OR disturb*) ADJ1 behav*) OR (behav* ADJ1 (aberration OR disorder* OR disturbance OR crisis)) OR runaway OR elopement OR "impulse control disorder*" OR "conduct disorder*" OR ((disruptive OR problem* OR dysfunctional) ADJ3 behav*) OR problembehav* OR impulsiv* OR impulsigenic OR disinhibition OR inhibition OR "sensation seek*" OR risk-taking OR "risk behav*" OR "risky behav*" OR irritability OR "irritable mood*" OR ((defiant OR hostile) ADJ1 behav*) OR aggress* OR counteraggress* OR anger OR "angry outbursts" OR provocation* OR deceitful* OR theft* OR stealing* OR ((antisocial OR anti-social OR asocial* OR deviant) ADJ3 (behav* OR conduct* OR personalit* OR reaction*)) OR "social behav* disorder*" OR misbehav* OR misconduct* OR delinquency OR delinquent* OR offender* OR callous-unemotional OR "callous and unemotional").mp.) AND (self-control/ OR self-regulation/ OR emotional regulation/ OR emotional control/ OR anger control/ OR executive function/ OR metacognition/ OR ego/ OR homeostasis/ OR cardiovascular reactivity/ OR heart rate/ OR baroreceptors/ OR autonomic nervous system/ OR (self-control OR "self manag*" OR "executive function*" OR "cognitive control" OR ((executive OR proactive OR reactive) ADJ1 control) OR "effortful control" OR metacognition OR metacognitive OR meta-cognit* OR ((emotion* OR affect) ADJ1 regulation) OR "emotional control" OR (control ADJ3 emotion*) OR "ego control" OR "ego undercontrol" OR "ego resiliency" OR "ego depletion" OR autoregulation OR self-regulat* OR homeostasis OR ((cardiac OR heart) ADJ1 (frequenc* OR rate*)) OR "respiratory sinus arrhythmia" OR "vagus tone" OR "vagal tone" OR vagotonus OR "vagus nerve tone" OR baroreflex* OR "pressoreceptor reflex" OR "baroceptor reflex" OR "baroreceptor reflex" OR "pressor reflex" OR "pressure reflex" OR "baroreceptor reflex" OR ((autonom* OR vegetative) ADJ3 system) OR ((adrenergic OR sympath* OR orthosympath*) ADJ3 (mechanism OR system))).mp.) AND (adolescent attitudes/ OR adolescent psychiatry/ OR adolescent psychology/ OR adolescent psychopathology/ OR adolescent psychotherapy/ OR exp juvenile delinquency/ OR (adolescent* OR adolescence OR preadolescen* OR pre-adolescen* OR teen* OR juvenile OR youth* OR "young adult*").mp.))

**Cochrane Library Wiley**

Cochrane Central Register of Controlled Trials, Issue 3 of 12, March 2021

72 references found, March 4, 2021

(((ecologic* NEXT momentary NEXT assess*) OR EMA):ab,ti,kw **AND** (externalizing OR externalising OR ODD OR ((oppositional OR defiant) NEXT/1 disorder*) OR "intermittent explosive disorder" OR (conduct NEXT disorder*) OR ((sociopath* OR psychopath* OR antisocial OR dyssocial) NEXT/1 (behav* OR personalit*)) OR sociopathy OR ((aberrant OR deviant OR disturb*) NEXT/1 behav*) OR (behav* NEXT/1 (aberration OR disorder* OR disturbance OR crisis)) OR runaway OR elopement OR ("impulse control" NEXT disorder*) OR (conduct NEXT disorder*) OR ((disruptive OR problem* OR dysfunctional) NEAR/3 behav*) OR problembehav* OR impulsiv* OR impulsigenic OR disinhibition OR inhibition OR (sensation NEXT seek*) OR risk-taking OR (risk NEXT behav*) OR (risky NEXT behav*) OR irritability OR (irritable NEXT mood*) OR ((defiant OR hostile) NEXT/1 behav*) OR aggress* OR counteraggress* OR anger OR "angry outbursts" OR provocation* OR deceitful* OR theft* OR stealing* OR ((antisocial OR anti-social OR asocial* OR deviant) NEAR/3 (behav* OR conduct* OR personalit* OR reaction*)) OR (social NEXT behav* NEXT disorder*) OR misbehav* OR misconduct* OR delinquency OR delinquent* OR offender* OR callous-unemotional OR "callous and unemotional"):ab,ti,kw **AND** (adolescent* OR adolescence OR preadolescen* OR (pre NEXT adolescen*) OR teen* OR juvenile OR youth* OR (young NEXT adult*)):ab,ti,kw) OR ((((experience NEXT sampling*) OR phenomenolog*):ab,ti,kw OR (("real time" OR (time NEXT course) OR ecological OR (in NEXT situ) OR naturalistic OR (real NEXT world) OR "daily life" OR "everyday life"):ab,ti,kw AND (experience* OR experiment* OR measur* OR questionnaire* OR interview* OR scale* OR (self NEXT report*) OR (self NEXT evaluation*) OR (self NEXT appraisal*) OR diary OR diaries OR assess* OR environment* OR approach*):ab,ti,kw) OR ((daily OR everyday) NEAR/3 (experience* OR experiment* OR measur* OR questionnaire* OR interview* OR scale* OR evaluation* OR appraisal* OR report* OR assess* OR diary OR diaries)):ab,ti,kw) AND (externalizing OR externalising OR ODD OR ((oppositional OR defiant) NEXT/1 disorder*) OR "intermittent explosive disorder" OR (conduct NEXT disorder*) OR ((sociopath* OR psychopath* OR antisocial OR dyssocial) NEXT/1 (behav* OR personalit*)) OR sociopathy OR ((aberrant OR deviant OR disturb*) NEXT/1 behav*) OR (behav* NEXT/1 (aberration OR disorder* OR disturbance OR crisis)) OR runaway OR elopement OR ("impulse control" NEXT disorder*) OR (conduct NEXT disorder*) OR ((disruptive OR problem* OR dysfunctional) NEAR/3 behav*) OR problembehav* OR impulsiv* OR impulsigenic OR disinhibition OR inhibition OR (sensation NEXT seek*) OR risk-taking OR (risk NEXT behav*) OR (risky NEXT behav*) OR irritability OR (irritable NEXT mood*) OR ((defiant OR hostile) NEXT/1 behav*) OR aggress* OR counteraggress* OR anger OR "angry outbursts" OR provocation* OR deceitful* OR theft* OR stealing* OR ((antisocial OR (anti NEXT social) OR asocial* OR deviant) NEAR/3 (behav* OR conduct* OR personalit* OR reaction*)) OR (social NEXT behav* NEXT disorder*) OR misbehav* OR misconduct* OR delinquency OR delinquent* OR offender* OR (callous NEXT unemotional) OR "callous and unemotional"):ab,ti,kw AND ((self NEXT control) OR (self NEXT manag*) OR (executive NEXT function*) OR "cognitive control" OR ((executive OR proactive OR reactive) NEXT/1 control) OR "effortful control" OR metacognition OR metacognitive OR (meta NEXT cognit*) OR ((emotion* OR affect) NEXT/1 regulation) OR "emotional control" OR (control NEXT/3 emotion*) OR "ego control" OR "ego undercontrol" OR "ego resiliency" OR "ego depletion" OR autoregulation OR (self NEXT regulat*) OR homeostasis OR ((cardiac OR heart) NEXT/1 (frequenc* OR rate*)) OR "respiratory sinus arrhythmia" OR "vagus tone" OR "vagal tone" OR vagotonus OR "vagus nerve tone" OR baroreflex* OR "pressoreceptor reflex" OR "baroceptor reflex" OR "baroreceptor reflex" OR "pressor reflex" OR "pressure reflex" OR "baroreceptor reflex" OR ((autonom* OR vegetative) NEXT/3 system) OR ((adrenergic OR sympath* OR orthosympath*) NEXT/3 (mechanism OR system))):ab,ti,kw AND (adolescent* OR adolescence OR preadolescen* OR (pre NEXT adolescen*) OR teen* OR juvenile OR youth* OR (young NEXT adult*)):ab,ti,kw)

**Web of Science – Core collection**

428 references found, March 4, 2021

(TS=(("ecologic* momentary assess*" OR "EMA") AND ("externalizing" OR "externalising" OR "ODD" OR (("oppositional" OR "defiant") NEAR/1 disorder*) OR "intermittent explosive disorder" OR "conduct disorder*" OR ((sociopath* OR psychopath* OR "antisocial" OR "dyssocial") NEAR/1 (behav* OR personalit*)) OR "sociopathy" OR (("aberrant" OR "deviant" OR disturb*) NEAR/1 behav*) OR (behav* NEAR/1 ("aberration" OR disorder* OR "disturbance" OR "crisis")) OR "runaway" OR "elopement" OR "impulse control disorder*" OR "conduct disorder*" OR (("disruptive" OR problem* OR "dysfunctional") NEAR/4 behav*) OR problembehav* OR impulsiv* OR "impulsigenic" OR "disinhibition" OR "inhibition" OR "sensation seek*" OR "risk-taking" OR "risk behav*" OR "risky behav*" OR "irritability" OR "irritable mood*" OR (("defiant" OR "hostile") NEAR/1 behav*) OR aggress* OR counteraggress* OR "anger" OR "angry outbursts" OR provocation* OR deceitful* OR theft* OR stealing* OR (("antisocial" OR "anti-social" OR asocial* OR deviant) NEAR/4 (behav* OR conduct* OR personalit* OR reaction*)) OR "social behav* disorder*" OR misbehav* OR misconduct* OR "delinquency" OR delinquent* OR offender* OR "callous-unemotional" OR "callous and unemotional") AND (adolescent* OR "adolescence" OR preadolescen* OR pre-adolescen* OR teen* OR "juvenile" OR youth* OR "young adult*") NOT (human* OR patient*))) OR (TS=(("experience sampling*" OR phenomenolog* OR (("real time" OR "time-course" OR "ecological" OR "in-situ" OR "naturalistic" OR "real-world" OR "daily life" OR "everyday life") AND (experience* OR experiment* OR measur* OR questionnaire* OR interview* OR scale* OR self-report* OR "self evaluation*" OR "self appraisal*" OR "diary" OR "diaries" OR assess* OR environment* OR approach*)) OR (("daily" OR "everyday") NEAR/3 (experience* OR experiment* OR measur* OR questionnaire* OR interview* OR scale* OR evaluation* OR appraisal* OR report* OR assess* OR "diary" OR "diaries"))) AND ("externalizing" OR "externalising" OR "ODD" OR (("oppositional" OR "defiant") NEAR/1 disorder*) OR "intermittent explosive disorder" OR "conduct disorder*" OR ((sociopath* OR psychopath* OR "antisocial" OR "dyssocial") NEAR/1 (behav* OR personalit*)) OR "sociopathy" OR (("aberrant" OR "deviant" OR disturb*) NEAR/1 behav*) OR (behav* NEAR/1 ("aberration" OR disorder* OR "disturbance" OR "crisis")) OR "runaway" OR "elopement" OR "impulse control disorder*" OR "conduct disorder*" OR (("disruptive" OR problem* OR "dysfunctional") NEAR/4 behav*) OR problembehav* OR impulsiv* OR "impulsigenic" OR "disinhibition" OR "inhibition" OR "sensation seek*" OR "risk-taking" OR "risk behav*" OR "risky behav*" OR "irritability" OR "irritable mood*" OR (("defiant" OR "hostile") NEAR/1 behav*) OR aggress* OR counteraggress* OR "anger" OR "angry outbursts" OR provocation* OR deceitful* OR theft* OR stealing* OR (("antisocial" OR "anti-social" OR asocial* OR "deviant") NEAR/4 (behav* OR conduct* OR personalit* OR reaction*)) OR "social behav* disorder*" OR misbehav* OR misconduct* OR "delinquency" OR delinquent* OR offender* OR "callous-unemotional" OR "callous and unemotional") AND ("self-control" OR "self manag*" OR "executive function*" OR "cognitive control" OR (("executive" OR "proactive" OR "reactive") NEAR/1 "control") OR "effortful control" OR "metacognition" OR "metacognitive" OR meta-cognit* OR ((emotion* OR "affect") NEAR/1 "regulation") OR "emotional control" OR ("control" NEAR/4 emotion*) OR "ego control" OR "ego undercontrol" OR "ego resiliency" OR "ego depletion" OR "autoregulation" OR self-regulat* OR "homeostasis" OR (("cardiac" OR "heart") NEAR/1 (frequenc* OR rate*)) OR "respiratory sinus arrhythmia" OR "vagus tone" OR "vagal tone" OR "vagotonus" OR "vagus nerve tone" OR baroreflex* OR "pressoreceptor reflex" OR "baroceptor reflex" OR "baroreceptor reflex" OR "pressor reflex" OR "pressure reflex" OR "baroreceptor reflex" OR ((autonom* OR "vegetative") NEAR/3 "system") OR (("adrenergic" OR sympath* OR orthosympath*) NEAR/4 ("mechanism" OR "system"))) AND (adolescent* OR "adolescence" OR preadolescen* OR pre-adolescen* OR teen* OR "juvenile" OR youth* OR "young adult*") NOT (human* OR patient*)))

Supplementary searches

**Google Scholar**

Search options, February 18, 2021

- Since 2017
- Do not include patents or citations
- 200 first results viewed

"ecological momentary assessment"|"ecological momentary assessments" externalising|externalizing symptom|disorder adolescent|teenager|youth|"young adult"|"young adults"

**Open grey**

<http://www.opengrey.eu/>

February 18, 2021

0 references found

ecologic* assessment* externali*

**ProQuest Dissertations & Theses A&I**

<https://search.proquest.com/pqdt/index>

37 references found, February 26, 2021

Search options

- Advanced search
- Search fields : Anywhere except full text - NOFT
- Doctoral dissertation only

noft("ecologic* momentary assess*" AND (adolescent* OR adolescence OR preadolescen* OR pre-adolescen* OR teen* OR juvenile OR youth* OR "young adult" OR "young adulthood" OR "young adults"))

**Dart Europe, Europe E-Theses Portal**

<https://www.dart-europe.org/basic-search.php>

32 references found, February 26, 2021

ecologic* momentary assess*

**LISSA**

<https://www.lissa.fr/dc/#env=lissa>

5 references found, February 25, 2021

Search options

- Advanced search
- Search fields : "Titre, mots-clés et résumé"

évaluation écologique instantanée OU évaluations écologiques instantanées

**SantéPsy (Ascodocpsy)**

<https://www.ascodocpsy.org/santepsy/>

1 reference found, February 25, 2021

évaluation* écologique* instantanée*

**ClinicalTrials.gov**

<https://clinicaltrials.gov/>

24 references found, February 18, 2021

ecological momentary assessment adolescent

**World health organization International clinical trials registry platform**

<https://apps.who.int/trialsearch/>

49 references found, February 18, 2021

ecological AND momentary AND assessment*
