# Supplementary material for: The added value of a micro-level ecological approach when mapping self-regulatory control processes and externalizing symptoms during adolescence: a systematic review
Source: Eur Child Adolesc Psychiatry. 2022 Mar 16;32(12):2387–97. doi: 10.1007/s00787-022-01972-1 (PMC10682160; doi:10.1007/s00787-022-01972-1)
Supplement: Supplementary file 2 — Supplementary file2 (DOCX 72 KB) [file 787_2022_1972_MOESM2_ESM.docx]

**Supplementary Table S1:**

| **Authors** | **# 1** | **#2** | **#3** | **#4** | **#5** | **#6** | **#7** | **#8** | **#9** | **#10** | **#11** | **#12** | **#13** | **#14** | **#15** | **#16** | **#17** | **#18** | **#19** | **#20** |
| --- | --- | --- | --- | --- | --- | --- | --- | --- | --- | --- | --- | --- | --- | --- | --- | --- | --- | --- | --- | --- |
| Beauchaine, Katkin, Strassberg, and Snarr (2001) | Yes | yes | Yes | Yes | Yes | Yes | No | Yes | Yes | Yes | Yes | Yes | Unknown | NA | Yes | Yes | Yes | Yes | No | Yes |
| Byrd et al. (2020) | Yes | Yes | No | Yes | Yes | Yes | No | Yes | Yes | Yes | Yes | Yes | Unknown | NA | Yes | Yes | Yes | Yes | No | Yes |
| Cui et al. (2019) | Yes | Yes | Yes | Yes | Yes | Yes | No | Yes | Yes | Yes | Yes | Yes | Unknown | NA | Yes | Yes | Yes | Yes | No | Yes |
| Cui et al. (2015) | Yes | Yes | No | Yes | Yes | Yes | No | Yes | Yes | Yes | Yes | Yes | Unknown | NA | Yes | Yes | Yes | Yes | No | Yes |
| de Ridder, Pihet, Suter, and Caldara (2016) | Yes | Yes | No | Yes | Yes | Yes | Yes | Yes | Yes | Yes | Yes | Yes | No | Yes | Yes | Yes | Yes | Yes | No | Yes |
| Diamond, Fagundes, and Cribbet (2012) | Yes | Yes | No | Yes | Yes | Yes | No | Yes | Yes | Yes | Yes | Yes | Unknown | NA | Yes | Yes | Yes | Yes | No | Yes |
| Gonzalez-Gadea et al. (2014) | Yes | Yes | No | Yes | Yes | Yes | No | Yes | Yes | Yes | Yes | Yes | Unknown | NA | Yes | Yes | Yes | Yes | No | Yes |
| Gunlicks-Stoessel and Powers (2008) | Yes | Yes | Yes | Yes | No | No | Yes | Yes | Yes | Yes | Yes | Yes | No | Yes | Yes | Yes | Yes | Yes | Don't know | Yes |
| Klahr et al. (2011) | Yes | Yes | No | Yes | Yes | Yes | No | Yes | Yes | Yes | Yes | Yes | Unknown | NA | Yes | Yes | Yes | Yes | No | Yes |
| Kuhn et al. (2018) | Yes | Yes | No | Yes | Yes | Yes | No | Yes | Yes | Yes | Yes | Yes | Unknown | NA | Yes | Yes | Yes | Yes | No | Yes |
| Maciejewski et al. (2018) | Yes | Yes | No | Yes | Yes | Yes | No | Yes | Yes | Yes | Yes | Yes | Unknown | NA | Yes | Yes | Yes | Yes | No | Yes |
| Moore, Hubbard, Bookhout, and Mlawer (2019) | Yes | Yes | No | Yes | Yes | Yes | No | Yes | Yes | Yes | Yes | Yes | Unknown | NA | Yes | Yes | Yes | Yes | No | Yes |
| Odgers and Russell (2017) | Yes | Yes | No | Yes | Yes | Yes | No | Yes | Yes | Yes | Yes | Yes | Unknown | NA | Yes | Yes | Yes | Yes | No | Yes |
| Pihet, De Ridder, and Suter (2017) | Yes | Yes | No | Yes | Yes | Yes | Yes | Yes | Yes | Yes | Yes | Yes | No | Yes | Yes | Yes | Yes | Yes | No | Yes |
| Rende, Slomkowski, Floro, and Jamner (2009) | Yes | Yes | No | Yes | Yes | Yes | No | Yes | Yes | Yes | Yes | Yes | Unknown | NA | Unknown | Yes | Yes | No | No | Yes |
| Rothenberg et al. (2019) | Yes | Yes | No | Yes | Yes | Yes | No | Yes | Yes | Yes | Yes | Yes | Unknown | NA | Yes | Yes | Yes | Yes | No | Yes |
| Santamaria-Garcia et al. (2019) | Yes | Yes | No | Yes | Yes | Yes | No | Yes | Yes | Yes | Yes | Yes | Unknown | NA | Yes | Yes | Yes | Yes | No | Yes |
| Schneiders et al. (2006) | Yes | Yes | No | Yes | Yes | Yes | No | Yes | Yes | Yes | Yes | Yes | Unknown | NA | Yes | Yes | Yes | Yes | No | Yes |
| Silk, Steinberg, and Morris (2003) | Yes | Yes | No | Yes | Yes | Yes | No | Yes | Yes | Yes | Yes | Yes | No | NA | Yes | Yes | Yes | Yes | No | Yes |
| Thomas et al. (2019) | Yes | Yes | No | Yes | Yes | Yes | No | Yes | Yes | Yes | Yes | Yes | Unknown | NA | Yes | Yes | Yes | Yes | No | Yes |
| Uink et al. (2018) | Yes | Yes | No | Yes | Yes | Yes | No | Yes | Yes | Yes | Yes | Yes | Unknown | NA | Yes | Yes | Yes | Yes | No | Yes |
| Uink, Modecki, and Barber (2017) | Yes | Yes | No | Yes | Yes | Yes | No | Yes | Yes | Yes | Yes | Yes | Unknown | NA | Yes | Yes | Yes | Yes | No | Yes |
| Ungvary et al. (2018) | Yes | Yes | No | Yes | Yes | Yes | Yes | Yes | Yes | Yes | Yes | Yes | No | Yes | Yes | Yes | Yes | Yes | No | Yes |
| Vannucci et al. (2018) | Yes | Yes | No | Yes | No | No | No | Yes | Yes | Yes | Yes | Yes | No | NA | Yes | Yes | Yes | Yes | No | Yes |

**Note.** NA: Not applicable. The list of items: Introduction: #1: Were the aims/objectives of the study clear?; Methods: #2: Was the study design appropriate for the stated aim(s)?; #3: Was the sample size justified?; #4: Was the target/reference population clearly defined? (Is it clear who the research was about?); #5: Was the sample frame taken from an appropriate population base so that it closely represented the target/reference population under investigation?; #6: Was the selection process likely to select subjects/participants that were representative of the target/reference population under investigation?; #7: Were measures undertaken to address and categorize non-responders?; #8: Were the risk factor and outcome variables measured appropriate to the aims of the study?; #9: Were the risk factor and outcome variables measured correctly using instruments/measurements that had been trialed, piloted or published previously?; #10: Is it clear what was used to determined statistical significance and/or precision estimates? (e.g., p values, CIs); #11: Were the methods (including statistical methods) sufficiently described to enable them to be repeated?; Results: #12: Were the basic data adequately described?; #13: Does the response rate raise concerns about non-response bias?; #14: If appropriate, was information about non-responders described?; #15: Were the results internally consistent?; #16: Were the results for the analyses described in the methods, presented?; Discussion: #17: Were the authors’ discussions and conclusions justified by the results?; #18: Were the limitations of the study discussed? Other: #19: Were there any funding sources or conflicts of interest that may affect the authors’ interpretation of the results? #20: Was ethical approval or consent of participants attained?

**Supplementary Figure S1:**

**Figure S1.** Summary of the AXIS assessment of the 28 studies included in the systematic review.

**References**

Beauchaine, T. P., Katkin, E. S., Strassberg, Z., & Snarr, J. (2001). Disinhibitory psychopathology in male adolescents: discriminating conduct disorder from attention-deficit/hyperactivity disorder through concurrent assessment of multiple autonomic states. *J Abnorm Psychol, 110*, 610-624.

Budziszewska, M., & Hansen, K. (2020). "Anger Detracts From Beauty": Gender Differences in Adolescents' Narratives About Anger. *Journal of Adolescent Research, 35*, 635-664.

Byrd, A. L., Vine, V., Beeney, J. E., Scott, L. N., Jennings, J. R., & Stepp, S. D. (2020). RSA reactivity to parent-child conflict as a predictor of dysregulated emotion and behavior in daily life. *Psychol Med*, 1-9.

Cui, L. X., Morris, A. S., Harrist, A. W., Larzelere, R. E., Criss, M. M., & Houltberg, B. J. (2015). Adolescent RSA Responses During an Anger Discussion Task: Relations to Emotion Regulation and Adjustment. *Emotion, 15*, 360-372.

Cui, L. X., Zhang, X. T., Houltberg, B. J., Criss, M. M., & Morris, A. S. (2019). RSA reactivity in response to viewing bullying film and adolescent social adjustment. *Developmental Psychobiology, 61*, 592-604.

de Ridder, J., Pihet, S., Suter, M., & Caldara, R. (2016). Empathy in Institutionalized Adolescents With Callous-Unemotional Traits: An Ecological Momentary Assessment Study of Emotion Recognition. *Criminal Justice and Behavior, 43*, 653-669.

Diamond, L. M., Fagundes, C. P., & Cribbet, M. R. (2012). Individual Differences in Adolescents' Sympathetic and Parasympathetic Functioning Moderate Associations Between Family Environment and Psychosocial Adjustment. *Developmental Psychology, 48*, 918-931.

Dirks, M. A., Treat, T. A., & Weersing, V. R. (2011). The Latent Structure of Youth Responses to Peer Provocation. *Journal of Psychopathology and Behavioral Assessment, 33*, 58-68.

Gonzalez-Gadea, M. L., Herrera, E., Parra, M., Mendez, P. G., Baez, S., Manes, F., & Ibanez, A. (2014). Emotion recognition and cognitive empathy deficits in adolescent offenders revealed by context-sensitive tasks. *Frontiers in Human Neuroscience, 8*.

Gunlicks-Stoessel, M. L., & Powers, S. I. (2008). Adolescents' Emotional Experiences of Mother-Adolescent Conflict Predict Internalizing and Externalizing Symptoms. *Journal of Research on Adolescence, 18*, 621-642.

Klahr, A. M., Rueter, M. A., McGue, M., Iacono, W. G., & Burt, S. A. (2011). The Relationship between Parent-Child Conflict and Adolescent Antisocial Behavior: Confirming Shared Environmental Mediation. *Journal of Abnormal Child Psychology, 39*, 683-694.

Kuhn, M. A., Ahles, J. J., Aldrich, J. T., Wielgus, M. D., & Mezulis, A. H. (2018). Physiological Self-Regulation Buffers the Relationship between Impulsivity and Externalizing Behaviors among Nonclinical Adolescents. *Journal of Youth and Adolescence, 47*, 829-841.

Maciejewski, D. F., Keijsers, L., van Lier, P. A. C., Branje, S. J. T., Meeus, W. H. J., & Koot, H. M. (2018). Most Fare Well—But Some Do Not: Distinct Profiles of Mood Variability Development and Their Association With Adjustment During Adolescence. *Developmental Psychology*.

Moore, C. C., Hubbard, J. A., Bookhout, M. K., & Mlawer, F. (2019). Relations between Reactive and Proactive Aggression and Daily Emotions in Adolescents. *Journal of Abnorm Child Psychology, 47*, 1495-1507.

Novin, S., & Rieffe, C. (2011). Anger Communication in Bicultural Adolescents. *Journal of Research on Adolescence, 22*, 80-88.

Odgers, C. L., & Russell, M. A. (2017). Violence exposure is associated with adolescents' same- and next-day mental health symptoms. *Journal of Child Psychology and Psychiatry, 58*, 1310-1318.

Pihet, S., De Ridder, J., & Suter, M. (2017). Ecological Momentary Assessment (EMA) Goes to Jail Capturing Daily Antisocial Behavior in its Context, a Feasibility and Reliability Study in Incarcerated Juvenile Offenders. *European Journal of Psychological Assessment, 33*, 87-96.

Rende, R., Slomkowski, C., Floro, J., & Jamner, L. (2009). Capturing Rule Breaking Behavior Between Siblings in Real Time and Everyday Settings. *European Journal of Developmental Science, 3*, 150-160.

Rothenberg, W. A., Di Giunta, L., Lansford, J. E., Lunetti, C., Fiasconaro, I., Basili, E., . . . Cirimele, F. (2019). Daily Associations between Emotions and Aggressive and Depressive Symptoms in Adolescence: The Mediating and Moderating Role of Emotion Dysregulation. *Journal of Youth and Adolescence, 48*, 2207-2221.

Santamaria-Garcia, H., Ibanez, A., Montano, S., Garcia, A. M., Patino-Saenz, M., Idarraga, C., . . . Baez, S. (2019). Out of Context, Beyond the Face: Neuroanatomical Pathways of Emotional Face-Body Language Integration in Adolescent Offenders. *Frontiers in Behavioral Neuroscience, 13*.

Schneiders, J., Nicolson, N. A., Berkhof, J., Feron, F. J., van Os, J., & Devries, M. W. (2006). Mood reactivity to daily negative events in early adolescence: Relationship to risk for psychopathology. *Developmental Psychology, 42*, 543-554.

Silk, J. S., Steinberg, L., & Morris, A. S. (2003). Adolescents' emotion regulation in daily life: Links to depressive symptoms and problem behavior. *Child Development, 74*, 1869-1880.

Thomas, S. A., Jain, A., Wilson, T., Deros, D. E., Jacobs, I., Dunn, E. J., . . . De Los Reyes, A. (2019). Moderated Mediation of the Link between Parent-Adolescent Conflict and Adolescent Risk-Taking: the Role of Physiological Regulation and Hostile Behavior in an Experimentally Controlled Investigation. *Journal of Psychopathology and Behavioral Assessment, 41*, 699-715.

Uink, B. N., Modecki, K. L., & Barber, B. L. (2017). Disadvantaged youth report less negative emotion to minor stressors when with peers: An experience sampling study. *International Journal of Behavioral Development, 41*, 41-51.

Uink, B. N., Modecki, K. L., Barber, B. L., & Correia, H. M. (2018). Socioeconomically Disadvantaged Adolescents with Elevated Externalizing Symptoms Show Heightened Emotion Reactivity to Daily Stress: An Experience Sampling Study. *Child Psychiatry & Human Development, 49*, 741-756.

Ungvary, S., McDonald, K. L., Gibson, C. E., Glenn, A. L., & Reijntjes, A. (2018). Victimized by Peers and Aggressive: The Moderating Role of Physiological Arousal and Reactivity. *Merrill-Palmer Quarterly-Journal of Developmental Psychology, 64*, 70-100.

Vannucci, A., Ohannessian, C. M., Flannery, K. M., De Los Reyes, A., & Liu, S. Q. (2018). Associations between friend conflict and affective states in the daily lives of adolescents. *Journal of Adolescence, 65*, 155-166.

Wang, L. S., & Sang, B. (2020). The Effect of Self-Regulation of Shame on Teenagers' Aggression. *Studia Psychologica, 62*, 58-73.
